# Development and psychometric evaluation of the mental readiness for military transition scale (MT-Ready)

**DOI:** 10.1186/s12888-023-05032-z

**Published:** 2023-08-08

**Authors:** Madeline Romaniuk, Gina Fisher, Matthew Sunderland, Philip J. Batterham

**Affiliations:** 1 Greenslopes Private Hospital, Gallipoli Medical Research Foundation, 121 Newdegate Street, 4120 Greenslopes, QLD Australia; 2 Faculty of Health and Behavioural Sciences, The University of Queensland, St Lucia, Australia; 3 Centre for Mental Health Research, National Centre for Epidemiology and Population Health, Australian National University, Canberra, Australia; 4 The Matilda Centre for Research in Mental health and Substance use, The University of Sydney, Camperdown, Australia

**Keywords:** Reintegration, Adjustment, Transition, Military, Defence, Psychometric development, Measure, Scale, Veteran, Mental health

## Abstract

**Background:**

The transition to civilian life following separation from military service is associated with increased risk of mental health disorders, suicide, and poor adjustment. No measure currently enables pre-separation screening to assess mental readiness for transition and identify personnel most at risk of poor outcomes. The Mental Readiness for Military Transition Scale (MT-Ready) was developed to identify psychosocial factors predictive of post-separation psychological adjustment and mental health.

**Methods:**

Phase I was a qualitative study including transitioned veterans (*n* = 60), partners of transitioned veterans (*n* = 20) and mental health clinicians (*n* = 20) which enabled development of candidate items that were subsequently piloted with a current serving Australian Defence Force (ADF) sample (*n* = 19). Phase II included evaluation of the factor structure, psychometric properties, and scale refinement of the initial pool of 50 items with a convenience sample of transitioning ADF personnel (*n* = 345). Analyses included exploratory factor analysis, evaluation of test-retest reliability, internal consistency, convergent, divergent, discriminant and predictive validity. Receiver Operating Characteristic Curve Analysis was also conducted to determine an optimal cut-off score.

**Results:**

Exploratory factor analysis resulted in a 15-item, three-factor solution that explained 62.2% of the variance: *Future focus and optimism; Anger and perceived failure; Civilian connections and social support*. Reliability and convergent, divergent, and discriminant validity was established. Receiver Operating Characteristic Curve Analysis determined a cut-off score of 55. MT-Ready scores significantly differentiated those reporting adjusting versus not adjusting to civilian life 3.7 months post-separation, and predicted post-separation outcomes including symptoms of Posttraumatic Stress Disorder, depression, anxiety, psychological adjustment and quality of life.

**Conclusions:**

This evaluation provides promising evidence the MT-Ready is a valid, reliable measure of mental readiness for transition, with predictive capability and considerable potential to assist prevention of poor post-separation outcomes among military personnel.

**Supplementary Information:**

The online version contains supplementary material available at 10.1186/s12888-023-05032-z.

## Background

Military transition refers to separation from military service, through voluntary or involuntary means, and moving from being a serving or active member of a Defence force to resuming a civilian life. More than an administrative process, separating from the military involves reconciling changes within individual, interpersonal, community, and societal domains [1]. Practical considerations and challenges of military transition often include re-location and securing housing, financial and healthcare changes, adjustments to children’s schooling and education, as well as securing new employment for both transitioning service members and their partners [2–5].

While many service members navigate transition and adjust well following separation, a substantial proportion report a difficult transition experience [6–9]. Australian research has found that approximately 78% of transitioned members experience a difficult transition out of military service, with 50% reporting they have not reintegrated or adjusted to civilian life up to 10 years after separation [6]. Canadian Armed Force members have reported comparable experiences with between 38% and 42% of transitioned members describing a difficult adjustment to civilian life [7, 8]. Similarly, US studies have found between 44% and 62% of transitioned members experience a difficult transition out of service [3, 9]. In light of reported difficulties, a growing body of research has highlighted the psychosocial challenges of transition and the subsequent impact these challenges may have on adjustment and reintegration post-service [7]. A systematic review examining transition across international military contexts found that a profound sense of loss of culture and community, identity, and purpose, were central themes related to psychological adjustment post-service [10]. Additional research has also identified psychosocial stressors during transition including adapting to new familial or marital dynamics [11, 12], navigating new social relationships and community connections [13], managing mental health conditions [14, 15], reconciling one’s sense of personal and social identity [7, 16] and re-adapting to aspects of civilian culture that may be at odds with military culture [2, 5, 17].

Military transition research has also demonstrated a concerning trend regarding the mental health of transitioned members [18–20]. A series of prevalence studies on Australian Defence Force (ADF) mental health examined a cohort of current serving/active duty and transitioned members [19, 21]. Compared to the current serving cohort, transtioned members reported significantly higher mental health difficulties including Posttraumatic Stress Disorder (PTSD), depressive and anxiety symptoms, alcohol use, suicidal ideation, as well as anger and general distress [19]. It was also estimated that 46% of the transitioned members (separated within the previous five years) met 12-month diagnostic criteria for a psychological condition using a structured interview, compared to 22% in the current serving cohort [19]. In addition to this research, national monitoring of suicide among current and former ADF members has consistently found that the rate of suicide among current serving members is significantly lower than the general Australian population, while the rate of suicide among transitioned members is significantly higher than the general Australian population [22]. Similar findings have also been reported in studies from the US and UK, indicating that as service members separate from the military, the rates of mental disorder and suicide sharply increase [18, 20, 23]. It may be possible that there is a vulnerability prior to transition for some members, which emerges as supports within Defence fall away upon separation. This suggests the period of transition carries both increased risk as well as the potential opportunity to embed preventative initiatives. Evidently, understanding how best to support military personnel through each stage of the transition process is critical for supporting both their immediate and long-term wellbeing. Focused efforts to assess members who are mentally ready for transition and members who are vulnerable to poor transition outcomes, prior to separation, may enable crucial opportunities to provide supportive interventions that could have a substantial impact on mental health trajectories over time.

While a number of measures have been developed to assess various aspects of reintegration outcomes [6, 24–27], these have been designed for use in veteran populations, post-separation. To the authors’ knowledge, two measures have been developed for intended use prior to military separation including a self-assessment tool [28] and a brief screener [8], both initiated by the Canadian Armed Forces/Department of National Defence and Veterans Affairs Canada.

The self-assessment tool was designed to capture a veteran’s subjective need for transition support services. Users consider a set of questions and rate themselves as one of three categories: Green (should be good to go), Yellow (think about it) and Red (consider seeking assistance) [28]. While certainly a useful tool to assist veterans considering if they need support during their transition to civilian life, it is not a psychometric measure and does not consider important psychosocial aspects such as sense of loss, potential apprehension about separation, or the presence of meaningful connections with civilians. The tool has not been validated with a current serving cohort prior to separation and it assumes that separation from military has already occurred as prompts/items include: “*Think about what life is like now that you have released”, “I have adjusted well to civilian life”* and *“my family has adjusted well to my transition to civilian life*” [28].

The brief screener prompts for potential difficulties in broad domains of health, mental health, need for and access to help, as well as satisfaction with activity and finances [8]. Again, while this screener may provide useful information for transition it similarly does not assess key psychosocial aspects (i.e. loss, identity, belonging, connections to civilians) and psychometric properties including predictive validity of outcomes (including adjustment to civilian life) has not been established or assessed. Further, the potential response to a number of items may be contingent on users’ membership in the military. For instance, satisfaction with main activity and financial situation are key items that may be endorsed positively based upon current employment within the military, which could potentially skew results/risk level for active-duty users.

There are currently no validated measures to assess service members’ mental readiness for transition to civilian life, to be used prior to separation, capturing key psychosocial determinants of adjustment and reintegration post-service. A scale that could indicate whether service members are likely to experience adjustment or reintegration difficulties prior to separation, specific to psychosocial factors, could facilitate early intervention or even prevention if linked with targeted, individualised support. The lack of empirical identification of psychosocial factors specifically relevant to current-serving members in transition may also inhibit the development of evidence-based interventions designed to mentally prepare at-risk service members for permanent separation.

### Aim

To address these limitations in current research and transition support services, a scale aimed at assessing mental readiness for permanent transition out of the military was developed. The Mental Readiness for Military Transition Scale, abbreviated to ‘MT-Ready’, was developed to facilitate prediction of adjustment and reintegration difficulties post-separation, and identify those at risk of poorer psychosocial outcomes post-service. This study describes the development process as well as an evaluation of the reliability and validity of the MT-Ready. The development process was undertaken in two phases: Phase I – Development of candidate items, and Phase II – Evaluation of the factor structure, psychometric properties, and scale refinement.

## Method

### Phase I – development of candidate items

The objective of Phase I was to use both inductive and deductive methods to develop a pool of candidate items for the MT-Ready. Phase I occurred as part of the development of the Military-Civilian Adjustment and Reintegration Measure (M-CARM) and detailed methods can be found pages 3–5 of Romaniuk et al. (2020) [6]. These methods are briefly summarized below.

A qualitative investigation of the lived experience of transitioned service members who had separated from the military was undertaken using interviews and focus groups with 60 transitioned Australian Defence Force (ADF) members, 20 partners of transitioned members, and 20 clinicians supporting current and former ADF members. Thematic analysis of the qualitative data as well as a systematic literature review [10] enabled the development of candidate items of the measure, focusing on themes related to those who mentally adjust well versus those who experience difficulty adjusting and reintegrating to civilian life. An expert panel review, comprised of clinicians and ADF service members, was then undertaken to appraise face validity, relevance and comprehensibility of the intended measure. A convenience sample of transitioning ADF members (*n* = 19) subsequently piloted 50 candidate items with the option of providing qualitative feedback on the items. All respondents found the 50 candidate items and Likert scale response options to be acceptable, and they were progressed to Phase II.

### Phase II – evaluation of factor structure, psychometric properties and scale refinement

Phase II of the study evaluated the MT-Ready in a sample of current-serving ADF personnel to determine the factor structure and assess the psychometric properties of the measure. Specific objectives included:

1. Determine the factor structure of the MT-Ready via Exploratory Factor Analysis.
2. Evaluate reliability of the MT-Ready via metrics of internal consistency and temporal stability.
3. Evaluate validity of the MT-Ready through convergent, divergent, and discriminant validity.
4. Evaluate predictive validity of the MT-Ready.
5. Determine and assess a screening cut-off score for the MT-Ready.

#### Participants

Participants were 345 current serving ADF members in the process of separating from military service. Participants were eligible if they were serving in the regular (active-duty) ADF, had submitted their Defence separation application form, or had received a notice of separation, and were not hospitalised for a psychological condition at the time of participation. A summary of participant demographic information is available in Table 1 as well as a comparison of available demographic information for the total ADF population, Table 2. The sample was predominately Male (75.3%), served in the Army (72.2%), from Other ranks (77.7%) with an average age of 36 years. While this was a convenience sample, age, gender and rank are comparable to the demographic breakdown of the total ADF (see Table 2). However, Army personnel and those involuntarily separating were overrepresented in the sample compared to the total ADF population. Participants were recruited through paid advertisements on social media sites as well as through on-base presentations and notices. Information about the study was also shared through the ADF intranet, and at ADF events such as transition seminars. The most consistent avenue of recruitment throughout the duration of the study was via the Soldier Recovery Centre, a rehabilitation centre based within Army barracks for injured military personnel, including those medically separating. This likely explains the overrepresentation of Army personnel involuntarily separating.

**Table 1:** Participant Demographic and Service Characteristics

Demographic variables	Full sample (*n* = 345)
Age, *M* (*SD*), range	36.15 (11.58), 19–61
Gender, % (*n*)	
Female	24.35 (84)
Male	75.36 (260)
Other	0.29 (1)
Ethnicity	
Caucasian	89.6 (309)
European	2.6 (9)
Asian	0.9 (3)
African	0.3 (1)
Middle Eastern	0.3 (1)
Aboriginal	2.9 (10)
Pacific Islander	0.6 (2)
Other	2.0 (10)
Marital status, % (*n*)	
Single	28.70 (99)
Married	40.58 (140)
Partner/De facto	30.72 (106)
Divorced, % (*n*)	18.26 (63)
Highest level of education, % (*n*)	
Primary	0.70 (3)
Secondary	38.26 (132)
TAFE	36.23 (125)
University	24.64 (85)
Service Type, % (*n*)	
Army	72.17 (249)
Navy	12.17 (42)
Air Force	15.65 (54)
Rank, % (*n*)	
Other ranks	77.68 (268)
Commissioned Officer	14.49 (50)
Warrant Officer	7.83 (27)
Discharge type, % (*n*)	
Voluntary	28.99 (100)
Medical	46.67 (161)
Administrative	16.8 (58)
Command initiated transition to reserves	3.77 (13)
Compulsory retirement age	3.77 (13)
Years of service, *M* (*SD*) range	13.58 (10.85), 1.08–43.92

**Table 2:** Participant characteristics in comparison to ADF statistics

Demographic Variables	Permanent ADF personnel* (*n* = 58,197)	Study Participants (*n* = 345)
Average age (*M*)	31	36
Gender, % (*n*)		
Male	79.90 (46,500)	75.36 (260)
Female	20.08 (11,687)	24.35 (84)
Did not disclose	-	0.29 (1)
Service Type, % (*n*)		
Army	48.78 (28,387)	72.17 (249)
Navy	25.70 (14,957)	12.17 (42)
Air Force	25.52 (14,853)	15.65 (54)
Rank, % (*n*)		
Other Ranks	72.27 (42,059)	77.68 (268)
Officers	27.73 (16,138)	22.32 (77)
Discharge type**, % (*n*)		
Voluntary	48.20 (2,733)	28.99 (100)
Involuntary***	49.47 (2805)	67.25 (232)
Retirement Age	2.33 (132)	3.77 (13)

*Note.* *Data for Permanent ADF personnel (*n* = 58,197) was obtained from the Defence Annual Report 2021-22. **Data for ADF separations (*n* = 5670) was obtained from the Defence Annual Report 2020-21. ***Includes Medical, Administrative, Command initiated transition to reserves and Trainee separations

#### Procedure

Ethical approval was obtained from the Department of Defence and Veterans’ Affairs Human Research Ethics Committee (DDVA HREC). In accordance with DDVA HREC procedures for recruiting currently serving ADF personnel, Command Approval was obtained from the Chief of the Defence Force (CDF) and Defence Organisational Support received by Joint Health Command who assessed the research merit and integrity as well as risk of the study. To determine eligibility, participants completed screening questions and were subsequently provided with detailed information about the study. Participants indicated consent online prior to proceeding to the survey via the Web Survey Creator platform. Data for the study was collected between March 2019 and October 2022.

Participants were invited to complete questionnaires at three timepoints. At timepoint 1, they completed all questionnaires described in the measures section. One week later, participants were asked to complete the MT-Ready a second time to examine test-retest reliability (timepoint 2). Participants were then emailed a web link to complete the timepoint 3 questionnaires (PCL-5, DASS-21, WHOQOL, M-CARM, Transition and adjustment questions) at least three months after their reported discharge date. Participants who had not yet separated, provided a revised date of discharge, and were re-contacted three months following the revised date.

A total of 1232 respondents completed screening questions at timepoint 1, with 469 respondents meeting eligibility based on self-report screening questions and providing consent to continue the survey. Reasons for ineligibility included not being in the ADF or serving in reserves only (*n* = 205), not separating from the ADF (*n* = 379), not having submitted discharge paperwork (*n* = 65), being currently hospitalised for a mental health condition (*n* = 31), not consenting to continue following participant information (*n* = 14) and exiting the survey at the screening point (*n* = 69). One hundred and seven participants commenced the survey but exited prior to completion of any measures. A further 17 responses were removed during data cleaning, due to duplicates identified and/or ineligibility. Of the remaining 345 participants who completed survey 1, 63% completed questionnaires at timepoint 2 (*n* = 217), and 42% completed questionnaires at timepoint 3 (*n* = 146). Eligibility for completion of timepoint 3 depended on discharge from the ADF, which varied between participants (i.e., not all were eligible to complete timepoint 3 within the study timeframe).

#### Measures

Table 3 presents descriptive statistics for all study measures including Mean, Range, Standard Deviation and Cronbach’s alpha.

**Table 3:** Descriptive statistics for all measures

Measure	*M*	*SD*	Range	Cronbach’s *a*
MT-Ready^1^	54.49	11.78	18–75	0.89
M2C-Q	22.17	15.85	0–64	0.95
WRFIS	33.67	12.95	14–70	0.93
PCL-5	28.63	21.45	0–80	0.97
COPE Inventory	122.30	21.06	62–187	0.91
DASS-21	15.33	20.08	0–63	0.97
WHOQOL	67.42	26.00	26–102	0.95
M-CARM^2^	69.2	16.64	28–105	0.92

*Note.*^1^MT-Ready *n* = 345. ^2^ M-CARM *n* = 146. Where not otherwise specified, *n* = 315

**Mental Readiness for Military Transition Scale (MT-Ready)**

The preliminary MT-Ready consisted of 50 items developed during Phase I of this study. To administer the scale, respondents are asked rate questions along a 5-point Likert scale (1 = *Disagree*, 2 = *Slightly Disagree*, 3 = *Neither Agree or Disagree*, 4 = *Slightly Agree*, 5 = *Agree*). It contains 6 reverse coded items. Higher scores indicate increased mental readiness for transition.

**Military-Civilian Adjustment and Reintegration Measure (M-CARM)**

The M-CARM [6, 10] is a 21-item self-report questionnaire assessing psychological and cultural reintegration to civilian life. The measure includes a number of key domains demonstrated to be important in healthy adjustment to civilian life following military service, among Australian ex-service personnel. Higher scores indicate healthier adjustment and reintegration. The measure has excellent psychometric properties [6, 10].

**PTSD Checklist for DSM-5 (PCL-5)**

The PCL-5 [29] is a 20-item self-report questionnaire that assesses the presence and severity of PTSD symptoms. The PCL-5 has demonstrated reliability and validity [30] and is used to assess symptom change over time, as well as screening individuals for a provisional diagnosis of PTSD. Provisional diagnosis was determined as per scoring guidelines [29] following the DSM-5 diagnostic rule which requires at least: 1 Criterion B item (questions 1–5), 1 Criterion C item (questions 6–7), 2 Criterion D items (questions 8–14), and 2 Criterion E items (questions 15–20), rated ‘Moderate’ or above. Higher scores indicate increased symptom severity.

**Depression Anxiety Stress Scale-21 (DASS-21)**

The DASS-21 [31] is a 21-item self-report questionnaire that assesses the degree of severity of depression, anxiety, and stress symptoms. The scale has shown good psychometric properties and is routinely used in research and clinical practice [31, 32]. Higher scores indicate increased symptom severity.

**The World Health Organization Quality of Life Instrument (WHOQOL)**

The brief WHOQOL [33] is a 26-item scale that assess quality of life across four broad domains: physical health, psychological health, social relationships, and environment. The brief WHOQOL has established validity [33] and higher scores indicate greater quality of life.

**Walter Reed Functional Impairment Scale (WRFIS)**

The Walter Reed Functional Impairment Scale [34] contains 14 items evaluating domains of functional impairment including social, physical, occupational and personal. This scale was developed for armed forces and has strong psychometric properties [34]. Higher scores indicate increased functional impairment.

**Military to Civilian Questionnaire (M2C-Q)**

The M2C-Q [35] is a self-report scale of post-deployment community reintegration difficulty, with 16 questions. It encompasses interpersonal connections, work, school or home productivity, engagement in the community, self-care, leisure activities, and overall sense of meaning. Previous research has indicated that the questionnaire possesses strong psychometric properties [35]. Higher scores indicate increased community reintegration difficulty.

**COPE Inventory (COPE)**

The COPE Inventory is a multidimensional coping questionnaire designed to assess various responses to stress. It measures aspects of ‘problem-focused’ coping (including planning), aspects of ‘emotion-focused’ coping (i.e., seeking of emotional social support); and other coping responses (i.e., venting of emotions, behavioural or mental disengagement). The questionnaire has established validity [36].

**Transition, Adjustment and Reintegration Questions**

At timepoint 1, participants were asked four questions reflecting self-reported preparation for transition including: (a) Do you think you are prepared for discharge? (Y/N), (b) Have you found the discharge process difficult? (Y/N), (c) Do you think you are prepared for your transition to civilian life? (Y/N), (d) Do you think you will easily reintegrate or adjust back to civilian life? (Y/N). At timepoint 3, participants were asked two questions reflecting overall post-separation adjustment and difficulty of transition including: (a) Did you find your transition out of the military difficult? (Y/N), (b) Do you think you have reintegrated or adjusted back to civilian life? (Y/N).

**Demographic Questionnaire**

Demographic characteristics, service details, and brief items regarding mental and physical health were also collected.

### Statistical analysis

Analyses were conducted using the IBM SPSS Version 28. For each variable, frequencies, descriptive statistics and graphs were produced and screened for outliers, and relevant assumptions. The measures did not reveal any instances of missing data or problematic outliers. Participants were able to conclude their participation in the web survey at any point before completing all the measures. As a result, 91% completed the survey in entirety (*n* = 315), while the remainder completed the MT Ready, as well as demographic and transition items but did not complete all additional measures (*n* = 30). Participants with incomplete data were removed listwise from validity analyses. There were no significant demographic differences between those who completed all measures versus those who did not. The MT-Ready collected at timepoint 1 was used for all analyses. Reverse-scored items were re-coded prior to analysis.

#### Factor analysis

The study utilized Exploratory Factor Analysis (EFA) with oblique rotation to explore the latent constructs and factor structure of the MT Ready. In order to ensure that the sample was adequate for analysis, the Kaiser-Meyer-Olkin value (which must be > 0.5) and the diagonal anti-image correlation matrix (which must be > 0.5) were examined. Additionally, Bartlett’s test of sphericity was analyzed to confirm intercorrelation between variables (which requires a p-value < 0.05). Inspection of Cattel’s scree plot and retaining only factors with an eigenvalue > 1 (Kaiser’s criterion) directed factor extraction. The number of factors retained was determined by (a) inspection of the scree plot (b) reviewing factor loadings and examining values < 0.50 (c) identifying factors with two items or less; and (d) conceptual reasoning and proposed application of the measure.

#### Reliability analysis

To assess the reliability of the measure, both its internal consistency and temporal stability were examined. Internal consistency was evaluated using Cronbach’s alpha, with an alpha value between 0.90 and 0.80 indicating excellent/good reliability, 0.79 to 0.70 indicating acceptable reliability, 0.69 to 0.60 indicating questionable reliability, 0.59 to 0.50 indicating poor reliability, and alpha values below 0.50 being considered unacceptable, with a recommended maximum alpha value of 0.90 [37]. Temporal stability was evaluated by computing the test-retest reliability coefficient on a subsample of participants (*n* = 206) who completed the measure twice, with the second administration taking place between seven and 14 days after the first. Both the total score and factor scores were examined for temporal stability using Pearson’s correlation, with higher coefficients indicating greater reliability (from 0 to + 1).

#### Validity analysis

Construct validity was evaluated through examination of convergent, divergent and discriminant validity metrics. To evaluate the direction and strength of the relationships between the total measure scores and other validated questionnaires related to mental health and functioning, Pearson’s correlation coefficients were calculated. Strength of association was interpreted using the following guideline: 0.50 or above = *strong*, 0.30 to 0.50 = *moderate*, 0.10 to 0.30 = *weak* [38]. Greater evidence of construct validity is shown by strong correlations with related constructs (convergent validity) and weaker correlations with unrelated constructs (divergent validity). Based on previous research showing a relationship between post-separation adjustment to civilian life and general functioning and mental health [6], it was predicted that the MT-Ready would strongly negatively correlate with the M2C-Q and at least moderately negatively correlate with the DASS-21 and WRFIS, and positively correlate with the WHOQOL.

To evaluate if the MT-Ready was able to differentiate between groups as theoretically expected, discriminant validity was examined. This involved performing *t*-tests to compare mean scores on the MT-Ready total score on four binary variables that were related to self-reported preparedness for transition, provisional PTSD diagnosis based on the PCL-5, and overall quality of life as rated by the WHOQOL global question (distinguishing ‘poor’/‘very poor’ from ‘good’/‘very good’).

Finally, predicative validity of the MT-Ready was also examined in a subset of participants (*n* = 144). A series of Pearson’s correlations were conducted with total scores of the MT-Ready collected before participants had separated from the ADF (timepoint 1) and measures of mental health and quality of life collected on average 3.7 (*range* = 3 to 11 months) after participants had separated from the ADF (timepoint 3). In addition, *t*-tests for mean differences on the MT-Ready total score from timepoint 1 were conducted as a function of two binary self-report variables reflecting overall post-separation adjustment and difficulty of transition (specified in the measures section), collected at timepoint 3. Effect sizes for *t*-test analyses using Cohen’s *d* were interpreted as follows: small (*d* = 0.2), medium (*d* = 0.5), and large (*d* = 0.8) [38]. Responses between timepoint 1 and timepoint 3 questionnaires were 9.5 months apart on average (*range* = 3.3 to 25.3 months).

#### Receiver operating characteristic (ROC) curve analysis

A ROC Curve analysis was conducted in order to determine an optimal cut-off point of readiness using sensitivity and specificity values to aid interpretation of scores and screen for potential adjustment and reintegration difficulties post-separation. The self-report binary question ‘Do you think you have reintegrated or adjusted back to civilian life? (Y/N)’ collected 3.7 months on average after separation was used as the outcome variable. Interpretation of the Area Under Curve (AUC) value included: > 0.90 = *very good*, > 0.80 = *good* and > 0.70 = *fair* [39]. Validation of the determined cut-off score included a series of *t*-tests comparing groups scoring above and below the cut-off on outcome measures including the PCL-5, DASS-21, M-CARM and WHOQOL collected 3.7 months on average after separation.

## Results

### Exploratory factor analysis

A Principal Axis Factoring analysis with oblique (non-orthogonal) rotation was conducted to explore latent constructs and factor structure of the measure. Sampling adequacy was verified by a Kaiser-Meyer-Olkin value of 0.945. In addition, all values on the diagonal anti-image correlation matrix were greater than 0.50. Bartlett’s test of sphericity demonstrated adequate intercorrelations between variables, *χ*^*2*^_1225_ = 8708.05, *p* < .001. Eleven factors were extracted with eigenvalues greater than one (1.01–16.47), explaining 63.67% of the variance. The point of inflection on Cattel’s scree plot suggested three factors. Inspection of the pattern matrix demonstrated a number of non-loading (loadings < 0.30), and relatively low-performing items (loadings < 0.40). A series of EFAs were run removing items with loadings < 0.30, followed by < 0.40, resulting in the removal of 14 items, then 13 items respectively. A further three items were removed with loadings < 0.50. Factors composed of two or less items were then removed (4 items). Each factor was then inspected for item redundancy to determine whether the overall length of the measure could be reduced. This was done in the context of application/future uses of the measure and consideration of survey fatigue among this population. Two factors each contained four items (the recommended number of items required to establish a valid factor), so psychometric properties of the remaining factor were reviewed as it contained eight items. It was identified that one item (23) could be removed from this factor without reducing the Cronbach’s alpha or overall explained variance. Conceptually, the item itself (“*the military doesn’t define who I am*”) appeared to be less related to the other items in the factor which were more indicative of focusing on the future than sense of identity (i.e. *“I’m looking forward to enjoying the freedoms of civilian life”, “I have thought carefully about, and planned my future out of the military”, “I am hopeful about my future outside of the military*”). As such, the item was removed, resulting in a more parsimonious solution of 15-items and 3-factors explaining 62.2% of the variance.

Factor 1, *Future-focus and optimism* contains seven items that assess members’ focus on their future and plans for life outside of the military, sense of readiness to leave the military and move on, and feelings of hope and optimism about the future including their ability to enjoy and adapt to civilian life. Factor 2, *Anger and perceived failure* contains four items that assess members’ anger at perceived treatment during service, including feeling ‘broken’ by the military, sense of failure within themselves and regrets about their service. Factor 3, *Civilian connections and social support* contains four items that assess members’ civilian friendships and connections through shared interests or beliefs, as well as presence of reliable support from family and friends. Factor correlations include: Factors 1 and 2, *r* = .34, Factors 1 and 3, *r* = .61, and Factors 2 and 3, *r* = .40. See Table 4 for item-total correlations, communalities, and factor loadings for the resulting solution. It contains 6 reverse coded items (denoted (R) in Table 4).

**Table 4:** Item–Total Correlations, Extracted Communalities and Oblique Rotated Three-Factor Solution for the MT-Ready (*n* = 345)

Item	Item-total correlation	*h* ^2^	Factors		
			1	2	3
I’m happy to leave the military behind and focus on other things.	0.501	0.532	0.858*	− 0.253	− 0.009
I feel ready to leave the military.	0.652	0.533	0.746*	0.105	− 0.029
I know I can adapt to the civilian way of life again.	0.670	0.551	0.666*	− 0.016	0.179
I’m looking forward to enjoying the freedoms of civilian life.	0.619	0.499	0.637*	− 0.001	0.129
I have thought carefully about, and planned my future out of the military.	0.631	0.453	0.594*	0.192	0.023
It will be hard for me to move on from my military service. (R)	0.596	0.431	0.570*	0.154	0.147
I am hopeful about my future outside of the military.	0.683	0.529	0.565*	0.166	0.024
I’m angry about the way I have been treated during my service. (R)	0.421	0.438	− 0.137	0.738*	0.106
I have a lot of regrets about my service. (R)	0.451	0.451	0.011	0.736*	− 0.015
The military broke me and is kicking me out. (R)	0.547	0.417	0.194	0.568*	0.030
I feel like a failure. (R)	0.624	0.482	0.292	0.566*	0.020
I have civilian friends.	0.506	0.448	0.037	− 0.112	0.736*
I have family members and/or friends who I can talk to when I need it.	0.595	0.510	0.071	0.020	0.699*
I’m not supported by friends and/or family. (R)	0.518	0.436	− 0.088	0.105	0.698*
Outside of the military, I have found people that I connect with through shared interests or beliefs.	0.545	0.427	0.097	0.021	0.586*
Variance explained (%)	62.20		41.70	11.98	8.51
Eigenvalues			6.26	1.80	1.28

*Note.* *Loadings > 0.50

Given the high proportion of involuntarily separated participants, an additional EFA was estimated to determine if a variation in factor structure was present for those who had voluntarily separated from the ADF. The three-factor structure was replicated in this subset, with all 15 items loading on the equivalent factors as the total sample.

### Reliability evaluation

Internal consistency was evaluated with the full sample (*n* = 345) and a Cronbach’s alpha of 0.89 was found for the total 15-item measure. Further, Cronbach alphas’ of 0.88, 0.79 and 0.79 were also found for Factors 1, 2 and 3 respectively. A subset of 206 participants completed the measure twice, between seven and 14 days apart. The 15-item measure total scores were strongly positively correlated between timepoint 1 and timepoint 2, *r* = .94 (*p* < .001). Each factor was also strongly correlated between timepoints: Factor 1, *r* = .91, Factor 2, *r* = .90, Factor 3, *r* = .82 (all *p* < .001).

### Validity evaluation

A subset of 315 participants (91% of total sample) completed all the additional measures to enable validity analysis.

#### Convergent validity

Correlations between total scores on the MT-Ready and the M2C-Q, WRFIS and PCL-5 as well as subscale scores on the DASS-21 and WHOQOL are presented in Table 5. Results demonstrated, as predicted, the MT-Ready total score was strongly negatively correlated with the M2C-Q the WRFIS the PCL-5 and the Depression, Anxiety and Stress DASS-21 subscales (all *p* < .001). Also as predicted, strong positive correlations were found between the total scores of the MT-Ready and each domain of the WHOQOL including Physical health, Psychological health, Social relationships, and Environment (all *p* < .001). Additional correlation analyses were undertaken for each factor of the MT-Ready and results are available in supplementary material.

**Table 5:** Correlations Between MT-Ready Scores and Validated Psychometric Measures Timepoint 1

Measure	1	2	3	4	5	6	7	9	10	11	12
1. MT-Ready Total	-										
2. M2C-Q Total	− 0.752	-									
3. WRFIS Total	− 0.615	0.766	-								
4. PCL-5 Total	− 0.639	0.819	0.717	-							
5. DASS-21 Depression	− 0.704	0.820	0.678	0.800	-						
6. DASS-21 Anxiety	− 0.500	0.732	0.626	0.799	0.759	-					
7. DASS-21 Stress	− 0.595	0.808	0.694	0.835	0.797	0.798	-				
9. WHOQOL Phys.	0.582	− 0.712	− 0.798	− 0.728	− 0.666	− 0.655	− 0.679	-			
10. WHOQOL Psych.	0.653	− 0.765	− 0.678	− 0.738	− 0.778	− 0.669	− 0.723	0.718	-		
11. WHOQOL Soc.	0.595	− 0.625	− 0.521	− 0.532	− 0.602	− 0.452	− 0.512	0.528	0.639	-	
12. WHOQOL Env.	0.631	− 0.701	− 0.646	− 0.657	− 0.626	− 0.587	− 0.636	0.712	0.697	0.655	-

*Note.* MT-Ready = Mental Readiness for Military Transition Scale; DASS-21 = Depression, Anxiety and Stress Scale-21; WRFIS = Walter Reed Functional Impairment Scale; WHOQOL Overall = World Health Organisation Quality of Life Scale Brief overall quality of life (item 2); WHOQOL Phys. = WHOQOL physical domain; WHOQOL Psych. = WHOQOL psychological domain; WHOQOL Soc. = WHOQOL social domain; WHOQOL Env = WHOQOL environmental domain

#### Divergent validity

Correlations between total scores on the MT-Ready and the COPE Inventory demonstrated lower associations relative to the above measures of mental health and functioning. For instance, coping subscales of behavioural disengagement (*r* = − .49), growth (*r* = .38), emotional social support (*r* = .34), planning (*r* = .33), active coping (*r* = .31), and acceptance (*r* = .31) exhibited moderate significant correlations (all *p* < .001), while coping subscales of humour (*r* = .10), religion (*r* = .12), and restraint (*r* = − .003) exhibited weak, non-significant correlations.

#### Discriminant validity

Mean scores for the MT-Ready and *t*-test results for binary transition preparedness variables, provisional PTSD diagnosis and quality of life are presented in Table 6. Scores on the MT-Ready were significantly different between groups on all transition preparedness/difficulties variables (all *p* < .001, *d* = 0.8 to 1.27). Those who met scoring criteria on the PCL-5 for provisional diagnosis of PTSD, also demonstrated significantly lower scores on the MT-Ready (*p* < .001, *d* = 1.12) as well as those who reported poor/very poor quality of life (*p* = < 0.001; *d* = -1.76; see Table 6).

**Table 6:** Mean MT-Ready Scores and *t*-test Results for Binary Variables Timepoint 1

	Yes				No				*t*-test				*Cohen’s d*
	*n*	*M*	*SD*		*n*	*M*	*SD*		*t*	*p*	*df*		
1. Prepared for discharge?	197	60.03	8.86		148	47.19	11.54		11.27	< 0.001	267*		1.27
2. Process difficult?	196	51.59	11.80		149	58.38	10.98		-5.46	< 0.001	343		-0.59
3. Prepared for transition?	193	60.63	8.90		152	46.77	10.72		13.11	< 0.001	343		1.42
4. Easily reintegrate?	197	60.62	8.80		148	46.40	10.64		13.56	< 0.001	343		1.23
Provisional PTSD diagnosis	182^2^	59.37	9.84		133	47.80	10.94		9.83	< 0.001	313		1.12
	Poor/Very poor				Good/Very good					*t*-test			*Cohen’s d*
	*n*	*M*	*SD*		*n*	*M*	*SD*		*t*	*p*	*df*		
Overall quality of life^2^	63	43.65	11.28		171	60.63	8.95		-10.76	< 0.001	92*		-1.76

*Note.* *Equal variances not assumed. Question 1: Do you think you are prepared for discharge? Question 2: Have you found the discharge process difficult? Question 3: Do you think you are prepared for your transition to civilian life? Question 4: Do you think you will easily reintegrate or adjust back to civilian life? Provisional PTSD diagnosis based on the PCL-5 criteria. Overall quality of life: Item 2 of the WHOQOL Brief. ^1^PCL-5 completed by n = 340. ^2^Analysis excluded neutral responses (3) to the WHOQOL global item (*n* = 232).

#### Predictive validity

Mean scores for the MT-Ready with *t*-test results for the two binary self-report questions determining subjective post-separation adjustment, reintegration and difficulty with transition are presented on Table 7. Scores on the MT-Ready collected at timepoint 1 (prior to separation) were significantly lower in those who reported they had not reintegrated or adjusted back to civilian life at timepoint 3 (on average 3.7 months following separation), *p* < .001, *d* = 1.15. In addition, scores on the MT-Ready collected at timepoint 1 were significantly lower in those who reported a difficult transition out of the military at timepoint 3, *p* < .001, *d* = -1.06.

**Table 7:** Mean MT-Ready Scores and *t*-test Results for Binary Variables Timepoint 3 (*n* = 146)

	Yes				No				*t*-test				*Cohen’s d*
	*n*	*M*	*SD*		*n*	*M*	*SD*		*t*	*p*	*df*		
1. Transition difficult?	77	44.32	12.00		69	60.70	9.03		-6.51	< 0.001	140*		-1.06
2. Adjusted or reintegrated?	74	60.63	10.39		72	48.59	10.62		6.92	< 0.001	144		1.15

*Note.* *Equal variances not assumed, Greenhouse Geisser correction applied. Question 1: Did you find your transition out of the military difficult? Question 2: Do you think you have reintegrated or adjusted back to civilian life?

Correlation coefficients between the MT-Ready collected timepoint 1 and measures of mental health and functioning collected timepoint 3 are presented Table 8. Strong significant negative correlations were found between the MT-Ready and post-separation outcome measures including the PCL-5, DASS-21 subscales of Depression, Anxiety, Stress, as well as strong significant positive correlations between WHOQOL domains of Physical health, Psychological health, Social relationships, and Environment, all *p* < .001. Additional correlation analyses were undertaken for each factor of the MT-Ready at timepoint 1 and measures collected at timepoint 3, and results are available in supplementary material.

**Table 8:** Correlations Between MT-Ready Scores and Outcome Measures Timepoint 3

Measure	1	2	3	4	5	6	7	8	9	
1. MT-Ready Total	-									
2. PCL-5 Total	− 0.586	-								
3. DASS-21 Depression	− 0.667	0.774	-							
4. DASS-21 Anxiety	− 0.551	0.777	0.779	-						
5. DASS-21 Stress	− 0.508	0.802	0.791	0.783	-					
6. WHOQOL Phys.	0.573	− 0.760	− 0.778	− 0.706	− 0.736	-				
7. WHOQOL Psych.	0.625	− 0.731	− 0.776	− 0.700	− 0.697	0.754	-			
8. WHOQOL Soc.	0.517	− 0.536	− 0.577	− 0.455	− 0.453	0.536	0.624	-		
9. WHOQOL Env.	0.497	− 0.637	− 0.618	− 0.567	− 0.521	0.654	0.667	0.593	-	

*Note.* MT-Ready = Mental Readiness for Military Transition Scale; DASS-21 = Depression, Anxiety and Stress Scale-21; WHOQOL Phys. = WHOQOL physical domain; WHOQOL Psych. = WHOQOL psychological domain; WHOQOL Soc. = WHOQOL social domain; WHOQOL Env = WHOQOL environmental domain. All *p*s < 0.001

#### Receiver operating characteristics (ROC) curve analysis

A ROC Curve analysis was conducted in order to determine an optimal cut-off point for the MT-Ready based on the Youden index (i.e., the highest balance of sensitivity and specificity). Analysis demonstrated good AUC = 0.81 [95% CI = 0.74-0.88], *p* < .001) and the cut-off value of 55 demonstrated sensitivity of 74% and specificity of 73%.

**Cut-off score validation**

Table 9 presents a series of *t*-tests comparing groups scoring above and below the MT-Ready cut-off on the PCL-5, DASS-21, M-CARM and WHOQOL collected post-separation. Results demonstrated significant differences in mean scores between groups on all measures (*p* < .001), with those scoring below the cut-off point (i.e., not mentally ready to transition) demonstrating significantly higher scores on the PCL-5 and DASS-21 and significantly lower scores on the M-CARM and WHOQOL 3.7 months on average after separation.

**Table 9:** MT-Ready cut-off groups and *t*-test Results for Binary Variables Timepoint 3

Time 3 Scores	< 55 (*n* = 67)			≥ 55 (*n* = 79)			*t*-test		*Cohen’s d*
	*M*	*SD*		*M*	*SD*		*t*	*df*	
M-CARM	57.84	11.64		80.18	12.95		-10.87	144	-1.81
PCL	35.76	21.90		16.91	17.33		5.68	125*	0.96
Depression	10.10	5.90		3.27	4.05		8.02	114*	1.37
Anxiety	7.15	5.26		2.09	3.27		6.84	107*	1.18
Stress	10.00	5.81		4.82	4.89		5.85	144	0.97
Environment	13.60	2.57		15.58	2.58		-4.62	144	-0.98
Physical health	10.86	3.38		14.82	3.17		-7.30	144	-1.21
Psychological health	10.01	3.64		14.40	3.76		-7.12	144	-1.18
Social health	10.31	3.56		13.96	3.73		-6.02	144	-1.00

*Note*. *Equal variances not assumed. All *p*s < 0.001

## Discussion

The MT-Ready was developed to address the lack of psychometric measures available to assess service members’ mental readiness for transition to civilian life, to be used prior to separation, capturing key psychosocial determinants of adjustment and reintegration post-service. This scale was developed using multi-phase methodology including a qualitative study of lived experience of transition, systematic literature review of psychological adjustment post-separation, expert panel review, pilot with intended users as well as empirical refinement of items using factor analysis. The resulting scale was then evaluated for validity and reliability using a number of well-established metrics.

### Factor structure

Following both inductive and deductive methods undertaken to generate items for the MT-Ready, a Principal Axis Factoring analysis was conducted resulting in a 15-item, 3-factor solution composed of the following factors: *Future-focus and optimism*, *Anger and perceived failure*, *Civilian connections and social support*. These non-clinical psychosocial factors were found to empirically contribute most to mental readiness for transition.

### Reliability and validity

Results demonstrated good internal consistency for the total score of the MT-Ready as well as each factor through high Cronbach’s alpha values. Temporal stability of the total score as well as each of the factors was excellent, with strong significant correlations found between timepoints.

Strong significant relationships were found in the predicted direction between the MT-Ready and measures of post-deployment community reintegration and functional impairment which were developed and validated for the military veteran population. Quality of life was also found to be significantly associated with the scale, with the ‘psychological health’ domain most strongly correlating with the MT-Ready. Given the intended use for assessing mental readiness specifically, these findings contribute further evidence of construct validity. The MT-Ready was also found to be significantly negatively associated with symptoms of PTSD, depression, anxiety and stress, indicating that as self-reported mental health symptoms increased, MT-Ready scores decreased. When examining the relationships between coping strategies and the MT-Ready, it was found that coping strategies of behavioural disengagement (e.g. avoiding problems) moderately correlated with reduced mental readiness, while strategies of growth, seeking emotional social support, planning, active coping and acceptance were moderately correlated with increased mental readiness. Finally, other coping strategies including use of humor and religion were not found to have any relationship with mental readiness for transition. Collectively these patterns of results give a sound indication of convergent and divergent validity. Findings are consistent with previous research linking psychological adjustment/reintegration to civilian life with mental health symptoms, quality of life and functioning within the veteran population [6, 34]. For instance, a prior study examining adjustment and reintegration within a veteran population, found similar strong and significant correlations with depression, anxiety, stress, quality of life, and functional impairment (i.e., *r* = .62 to 0.76) [10]. Findings also revealed comparatively weaker correlations with constructs in which weaker associations would be expected based on previous evidence, such as coping through humour and/or religious beliefs [40].

Sound evidence of discriminant validity was established, with the MT-Ready able to significantly distinguish between groups by reported transition preparedness, PTSD and quality of life. Results revealed that scores on the MT-Ready were significantly lower among those who reported they: (1) were not prepared for discharge, (2) had found the discharge process difficult, (3) were not prepared for transition to civilian life, and (4) did not think they would easily adjust or reintegrate back to civilian life. Further, those with a provisional diagnosis of PTSD also demonstrated significantly lower scores on the MT-Ready. Finally, those who reported a global rating of poor/very poor quality of life exhibited significantly lower scores on the scale compared to those who reported good/very good quality of life.

Initial evidence of predictive validity was also found. Results indicated those who reported they had not reintegrated or adjusted back to civilian life between three and 11 months (*M* = 3.7 months) after separation, demonstrated significantly lower scores on the MT-Ready completed prior to separation, with a large effect size. Results also demonstrated those who reported a difficult transition out of the military between three and 11 months after separation, had significantly lower scores on the MT-Ready completed prior to separation, with a large effect size. Further, responses on the MT-Ready collected prior to separation strongly and significantly negatively correlated with self-reported symptoms of PTSD, depression, anxiety, and stress 3.7 months on average after separation. Reported quality of life 3.7 months on average after separation was strongly and significantly correlated with responses on the MT-Ready collected prior to separation. This indicates that lower scores on the MT-Ready may be predictive of decreased quality of life and increased mental health difficulties following separation, however this evidence is preliminary.

Finally, following ROC curve analysis establishing an optimal cut-off point for the MT-Ready, it was found that members who scored on or above the cut-off of 55 prior to separation (i.e. mentally ready to transition) demonstrated significantly lower symptoms of PTSD, depression, anxiety and stress as well as significantly higher psychological adjustment, cultural reintegration and quality of life 3.7 months on average after separation, compared to members who scored below the cut-off point.

### Initial scoring and interpretation

To score the MT-Ready, six items are reversed-scored and a total score is derived from summing the 15-items. Scores range from 15 to 75, with higher scores indicating increased mental readiness, specific to the three MT-Ready factors. A score of 55 and above indicates a member is likely to have reported higher levels of future-focus, optimism, civilian connections and social support and lower levers of anger and perceived failure. A score below 55 indicates a member is more likely to require further support and has an increased risk of adjustment and reintegration difficulties post-separation. In addition, a scoring profile may be created by calculating an average score for each factor as a subscale, and plotting values in relation to other subscale scores. Lower subscale scores (with an average of three or below) may indicate focus points to assist psychosocial treatment planning and sequencing or prioritizing of transition support.

Due to the limitations/error inherent when conducting ROC curve analysis, the suggested cut-off score should always be interpreted in conjunction with each factor in order to help determine tiered transition support ‘pathways’ to facilitate ideal levels of support. For instance, a member might score above the cut-off, indicating increased likelihood of adjustment post-separation, however a lower scoring domain may be indicated (i.e. an average factor score of three or lower) and as such, the opportunity to provide additional support or resources in this area should be considered. Table 10 provides an initial interpretation guide for scores on the MT-Ready, noting this is a guide only and not intended to be prescriptive or used in isolation of comprehensive/additional relevant information about a transitioning member.

**Table 10:** MT-Ready Scoring Interpretation Guide

Transition Pathway	MT-Ready Total Score	Number of Factors scoring 3 or lower	Support level	% (*n*) in the current sample
Pathway 1: Guided and collaborative	Below 55	2 or more	The member is supported in accessing psychosocial care in key areas of need identified by the MT-Ready with a mental health professional.	24.1 (83)
Pathway 2: Self-managed with resources and follow-up provided	Below 55	1 or less	The member is provided with options and resources to access psychosocial support in key areas of need with a mental health professional or more generally via a support organisation. Follow-up is also provided.	23.2 (80)
Pathway 3: Self-managed with resources provided	55 or above	1 or more	The member is provided with options and resources to access psychosocial support in key areas of need with a mental health professional or more generally via a support organisation.	14.8 (51)
Pathway 4: Self-managed	55 or above	0	The member is unlikely to require additional psychosocial support.	38.0 (131)

There is no clear evidence from past research regarding the optimal time to assess for transition readiness, mentally or otherwise. That said, given the overarching aim of taking a preventative approach to poor adjustment, it would be reasonable to suggest that completion of the scale occurs at the earliest feasible timepoint once separation is determined. This may vary between members, but the crucial consideration would be allowing time to refer to appropriate transition pathways and access the optimal level of support prior to separation. Finally, while the validity evidence suggests scores on the MT-Ready are associated with mental health symptoms, the scale is not a diagnostic clinical instrument. As such, if mental health concerns are indicated, the MT-Ready should be used in conjunction with validated psychometric mental health measures and/or clinical interview.

### Implications of results

Overall, the findings of this study contribute towards evidence that the MT-Ready is a valid and reliable tool with predictive capability to aid the assessment of mental readiness for permanent transition out of military service. The scale enables identification of the non-clinical psychosocial factors that contribute most to mental readiness. An evidence-based tool that facilitates individualised assessment of readiness for transition, including identification of specific psychosocial needs impacting readiness, is a crucial step towards a proactive preventative or ‘prehabilitation’ approach to adjustment and reintegration to civilian life. Addressing this gap is vital, given the substantial evidence indicating transition out of military service is associated with increased risk of the development of psychological disorders as well as suicide [18–20, 22, 23]. While understanding and identifying those most ‘at risk’ for psychological adjustment and reintegration difficulties at a population level is essential, assessing mental readiness (and reasons for lack of readiness) at an individual level has the potential to advance the development of bespoke transition support pathways for every ‘at risk’ member.

The MT-Ready has potential for both further research and clinical application within the serving military population, as there are currently no other measures that have been developed within this group and designed specifically to assess mental readiness for permanent separation from the military. The scale may enable novel research regarding mental readiness trajectories over time, throughout military service, and the potential impact on mental health and functioning. This may be particularly useful for supporting personnel who face an unexpected involuntary/medical discharge, a group that is consistently found to be most at risk of poorer outcomes post-service across longitudinal and cross-sectional research [41, 42].

In addition to possible implementation within Defence, the MT-Ready may be useful within clinical and community services that offer adjunct transition support to current serving members by providing an indication of mental readiness and identifying potential intervention pathways to improve readiness and consequently adjustment post-service. There are specific therapeutic modalities or strategies that may be useful in targeting each factor of the MT-Ready. For instance, ‘learned optimism’, part of the Positive Psychology framework [43], provides a model in which optimism can be cultivated through evidence-based cognitive-behavioural strategies. Anger and feelings of failure are emotional experiences that can have various determinants or maintaining factors, but may also shift with cognitive-behavioural approaches. Assessment and treatment planning with a psychologist/mental health clinician may illuminate treatment targets driving problematic anger or feelings of failure such as unhelpful thinking patterns (for example, preoccupation with regrets from service), high levels of self-criticism or shame, or difficulties processing and managing intense emotions. Techniques from Cognitive Behavioural Therapy (e.g., cognitive challenging, emotion regulation strategies), Acceptance and Commitment Therapy (e.g., defusion, mindfulness, distress tolerance strategies), and Compassion Focused Therapy (e.g., strategies for cultivating compassion toward self), each have evidence supporting effectiveness in improving such treatment targets [44–46].

In relation to social support and civilian connections, there are numerous avenues for service members and veterans to engage in support services and programs aimed at improving social connection and engagement [47]. These may be through Ex-Service Organisations (ESOs), government services as well as through rehabilitation case managers who connect service members into community activities, groups and organisations with aligned interests and values. Addressing psychological barriers to engagement and social connection may also be an important consideration, including addressing cultural differences with civilians, as well as mental health symptoms contributing to potential isolation and withdrawal.

### Limitations & future directions

There are a number of limitations to address in this study. First, the sample included a high proportion of personnel flagged for involuntary separation. While the factor structure was replicated within the voluntary group, an additional validation study using a larger sample more representative of the Defence population would be valuable. A representative sample would also allow for the development of normative data that may inform more nuanced decisions around support pathways. Recruitment methods would need to be carefully considered in order to prevent overrepresentation within a particular sub-group. Second, a replication of the factor structure through Confirmatory Factor Analysis would contribute further to the validity of this scale as well as establish evidence of a potential bi-factor model. This could also include multigroup CFA to test measurement invariance across participants from various groups (i.e. gender, rank, length of service), which was not possible in this study due to sample size limitations. Third, predictive validity was initially assessed in this study, however the optimal cut-off score was determined using a single self-report item. Future research could progress by extending the longitudinal follow-up timepoints as well as including objective or behavioural indicators of adjustment and reintegration in addition to self-report. Using self-report and convenience sampling methods may raise the possibility of participant bias, such as social desirability and self-selection. Additionally, service status was not verified with Defence records in this study. Finally, future research assessing the scale’s sensitivity to detect change over time would also be worthwhile as well as cross-cultural validation for application within other Defence Forces.

## Conclusion

The MT-Ready is the first psychometric measure developed with a currently serving military population, designed to assess mental readiness for permanent separation from the military. Developed using both inductive and deductive methods, EFA resulted in a 3-factor structure including Factor 1, *Future-focus and optimism*, Factor 2, *Anger and perceived failure* and Factor 3, *Civilian connections and social support.* Psychometric evaluation of the MT-Ready demonstrated strong reliability and construct validity including convergent, divergent, and discriminant validity as well as internal consistency and temporal stability. Preliminary evidence of predictive validity was also found. Members who reported difficulties adjusting to civilian life following separation had reported significantly lower MT-Ready scores before separation. Further, MT-Ready scores obtained prior to separation strongly and significantly correlated with quality of life and mental health symptoms 3.7 months after separation. The MT-Ready has 15 self-report items and takes approximately 2 minutes to complete. ROC curve analysis established a cut-off point of 55, indicating those scoring below this may have an increased risk of difficulty adjusting to civilian life. While further evaluation of the scale would be ideal, the MT-Ready may be a useful tool to assist in the prevention of poor psychosocial outcomes among military personnel after permanent separation from service.

### Electronic supplementary material

Below is the link to the electronic supplementary material.

**Supplementary Table 1**: Correlations Between MT-Ready Factor Scores and Validated Psychometric Measures - Timepoint 1. **Supplementary Table 2**: Correlations Between MT-Ready Factor Scores, and COPE Inventory - Timepoint 1. **Supplementary Table 3**: Correlations Between MT-Ready Factor Scores at Timepoint 1, and Validated Psychometric Measures Timepoint 3. **Supplementary Table 4**: Candidate items for the Mental Readiness for Military Transition Scale (MT-Ready). © Romaniuk, M. (2018) Gallipoli Medical Research Foundation.

## Data Availability

The data that support the findings of this study are available from Gallipoli Medical Research Foundation, but restrictions apply to the availability of these data, as it pertains to currently serving military personnel, and so are not publicly available. Data are however available from the authors upon reasonable request to principal investigator Dr Madeline Romaniuk and with permission from DDVA HREC.

## List of abbreviations

ADF: Australian Defence Force
DDVA HREC: The Department of Defence and Veterans’ Affairs Human Research Ethics Committee
CDF: Chief of the Defence Force
COPE: COPE Inventory
DASS-21: Depression Anxiety Stress Scale-21
EFA: Exploratory Factor Analysis
M2C-Q: Military to Civilian Questionnaire
MT-Ready: Mental Readiness for Military Transition Scale
M-CARM: Military-Civilian Adjustment and Reintegration Measure
PCL-5: PTSD Checklist for DSM-5
PTSD: Posttraumatic Stress Disorder
ROC: Receiver Operating Characteristic
WHOQOL-BREF: The World Health Organization Quality of Life Instrument
WRFIS: Walter Reed Functional Impairment Scale
