# Supplementary material for: Development and psychometric evaluation of the mental readiness for military transition scale (MT-Ready)
Source: BMC Psychiatry. 2023 Aug 8;23:575. doi: 10.1186/s12888-023-05032-z (PMC10410831; doi:10.1186/s12888-023-05032-z)
Supplement: Supplementary file 1 — Supplementary Table 1: Correlations Between MT-Ready Factor Scores and Validated Psychometric Measures - Timepoint 1. Supplementary Table 2: Correlations Between MT-Ready Factor Scores, and COPE Inventory - Timepoint 1. Supplementary Table 3: Correlations Between MT-Ready Factor Scores at Timepoint 1, and Validated Psychometric Measures Timepoint 3. Supplementary Table 4: Candidate items for the Mental Readiness for Military Transition Scale (MT-Ready). © Romaniuk, M. (2018) Gallipoli Medical Research Foundation. [file 12888_2023_5032_MOESM1_ESM.pdf]

Supplementary Table 1

Correlations Between MT-Ready Factor Scores and Validated Psychometric Measures - Timepoint 1

Measure	1	2	3	4	5	6	7	8	9	10	11	12	13	14
1. MT-Ready Total	-													
2. MT-Ready Factor 1	.885	-												
3. MT-Ready Factor 2	.762	.464	-											
4. MT-Ready Factor 3	.759	.571	.388	-										
5. M2C-Q Total	-.752	-.590	-.680	-.558	-									
6. WRFIS Total	-.615	-.467	-.638	-.376	.717	-								
7. PCL-5 Total	-.639	-.477	-.669	-.396	.678	.800	-							
8. DASS-21 Depression	-.704	-.544	-.669	-.495	.626	.799	.800	-						
9. DASS-21 Anxiety	-.500	-.378	-.530	-.295	.694	.835	.799	.759	-					
10. DASS-21 Stress	-.595	-.446	-.601	-.395	-.798	-.728	.835	.797	.798	-				
11. WHO-QOL Phys.	.582	.437	.657	.292	-.678	-.738	-.728	-.666	-.655	-.679	-			
12. WHO-QOL Psych.	.653	.486	.653	.450	-.521	-.532	-.738	-.778	-.669	-.723	.718	-		
13. WHO-QOL Soc.	.595	.443	.485	.554	-.646	-.657	-.532	-.602	-.452	-.512	.528	.639	-	
14. WHO-QOL Env.	.631	.464	.623	.455	.717	.800	-.657	-.626	-.587	-.636	.712	.697	.655	-

-*Note.* MT-Ready = Mental Readiness for Military Transition Scale; DASS-21 = Depression, Anxiety and Stress Scale-21; WRFIS = Walter Reed Functional Impairment Scale; WHO-QOL Overall = World Health Organisation Quality of Life Scale Brief overall quality of life (item 2); WHO-QOL Phys. = WHO-QOL physical domain; WHO-QOL Psych. = WHO-QOL psychological domain; WHO-QOL Soc. = WHO-QOL social domain; WHO-QOL Env = WHO-QOL environmental domain.

Supplementary Table 2

Correlations Between MT-Ready Factor Scores, and COPE Inventory - Timepoint 1

Measure	1	2	3	4	5	6	7	8	9	10	11	12	13	14
1. MT-Ready Total	-													
2. MT-Ready Factor 1	.885	-												
3. MT-Ready Factor 2	.762	.464***	-											
4. MT-Ready Factor 3	.759	.571***	.388	-										
5. Behavioural disengagement	-.490***	-.408***	-.432***	-.339***	-									
6. Growth	.384***	.345***	.294***	.281***	-.257***	-								
7. Emotional	.339***	.292***	.211***	.334***	-.124*	.454***	-							
8. Social	.325***	.302***	.207***	.276***	-.121*	.577***	.701***	-						
9. Planning	.333***	.316***	.255***	.212***	-.248***	.738***	.389***	.565***	-					
10. Active Coping	.318***	.299***	.254***	.194**	-.239***	.713***	.440***	.640***	.818***	-				
11. Acceptance	.308***	.261***	.275***	.200***	-.104	.590***	.236***	.369***	.566***	.539***	-			
12. Humour	.103	.159**	.014	.045	.063	.364***	.122*	.237***	.358***	.346***	.350***	-		
13. Religion	.113*	.092	.076	.113*	-.033	.132*	.135*	.082	.084	.095	-.014	.042	-	
14. Restraint	-.001	.005	.010	-.046	.165**	.316***	.221***	.351***	.381***	.375***	.401***	.198***	.021	-

Note. MT-Ready = Mental Readiness for Military Transition Scale; COPE = Coping Orientation to Problems Experienced Inventory Total. *p <.05; **p <.01 ****p* < .001.

Supplementary Table 3

Correlations Between MT-Ready Factor Scores at Timepoint 1, and Validated Psychometric Measures Timepoint 3

Measure	1	2	3	4	5	6	7	8	9	10	11	12	13
1. MT-Ready Total	-												
2. MT-Ready Factor 1	.885**	-											
3. MT-Ready Factor 2	.762**	.457**	-										
4. MT-Ready Factor 3	.744**	.572**	.364**	-									
5. PCL-5 Total	-.586**	-.432**	-.617**	-.348**	-								
6. DASS-21 Depression	-.667**	-.504**	-.699**	-.379**	.774**	-							
7. DASS-21 Anxiety	-.551**	-.463**	-.547**	-.273*	.777**	.779**	-						
8. DASS-21 Stress	-.508**	-.372**	-.598**	-.219*	.802**	.791**	.783**	-					
9. WHO-QOL Phys.	.573**	.439**	.653**	.238*	-.760**	-.778**	-.706**	-.736**	-				
10. WHO-QOL Psych.	.625**	.453**	.651**	.396**	-.731**	-.776**	-.700**	-.697**	.754**	-			
11. WHO-QOL Soc.	.517**	.337**	.497**	.457**	-.536**	-.577**	-.455**	-.453**	.536**	.624**	-		
12. WHO-QOL Env.	.497**	.362**	.505**	.331**	-.637**	-.618**	-.567**	-.521**	.654**	.667**	.593**	-	
13. M-CARM	.773**	.623**	.697**	.532**	-.704**	-.762**	-.630**	-.671**	.711**	.739**	.672**	.625**	-

Note. MT-Ready = Mental Readiness for Military Transition Scale; DASS-21 = Depression, Anxiety and Stress Scale-21; WHO-QOL Phys. = WHO-QOL physical domain; WHO-QOL Psych. = WHO-QOL psychological domain; WHO-QOL Soc. = WHO-QOL social domain; WHO-QOL Env = WHO-QOL environmental domain. M-CARM = Military-Civilian Adjustment and Reintegration Measure. *p <.01 **p < .001

Supplementary Table 4

Candidate items for the Mental Readiness for Military Transition Scale (MT-Ready)
© Romaniuk, M. (2018) Gallipoli Medical Research Foundation.

1. I know how to access professional support for my health.
2. I have thought about what it will feel like to not put the uniform on anymore.
3. I am hopeful about my future outside the military.
4. I have nothing in common with civilians. **R**
5. I am a flexible person and I don't mind changing to suit others when required.
6. I won't have direction when I get out. **R**
7. I feel valued by others whether I'm in or out of the military.
8. My military service is an important chapter in my life, but there are more chapters to come.
9. I have family members and/or friends who I can talk to when I need it.
10. I feel I have nothing left now that my service is ending. **R**
11. When in a civilian setting I know I need to adapt myself to fit into that environment.
12. I'm angry about the way I have been treated during my service. **R**
13. People should behave like I do. **R**
14. Outside of the military, I have found people that I connect with through shared interests or beliefs.
15. Civilians are disrespectful and rude. **R**

16. When people frustrate me I have ways to help me remain calm.
17. I don't think society puts much value on military service and experience. **R**
18. I feel like my best days are behind me. **R**
19. I have civilian friends.
20. The military broke me and is kicking me out. **R**
21. I would never seek help from a mental health professional. **R**
22. I'm looking forward to enjoying the freedoms of civilian life.
23. The military doesn't define who I am.
24. I tend to only associate with other people who are or were in the military. **R**
25. I don't think I will ever be able to adapt to a civilian mindset. **R**
26. I know how I will occupy my time productively when I get out.
27. I'm happy to leave the military behind and focus on other things.
28. When I'm having a bad day there are a few strategies I can use to get through it.
29. I try and drop my 'military speak' when I'm around civilians.
30. I have interests and hobbies outside of the military that are enjoyable or meaningful.
31. When I leave the military, I will lose a lot and not gain much. **R**
32. I find it difficult to change once I have a set routine. **R**
33. I know I can adapt to the civilian way of life again.

34. I would ask for help if I needed it.
35. I feel like a failure. **R**
36. Civilians seem to be concerned with trivial matters. **R**
37. I know who I am whether I wear a military uniform or not.
38. I have thought carefully about, and planned my future out of the military.
39. It will be hard for me to move on from my military service. **R**
40. I find it difficult to ask for help if I'm struggling. **R**
41. I have a lot of regrets about my service. **R**
42. I am more regimented than flexible. **R**
43. I have things that give me a sense of achievement, outside of paid employment.
44. I think civilians will respect my rank and military experience in the workplace. **R**
45. I'm not supported by friends and/or family. **R**
46. I will lose my life purpose when I discharge. **R**
47. I feel like I've had some control over my discharge.
48. I expect to easily find a good job once I'm out. **R**
49. I don't know how to manage my frustrations. **R**
50. I feel ready to leave the military.
